# Hepatocellular carcinoma surveillance: current practice and future directions

**DOI:** 10.20517/2394-5079.2021.131

**Published:** 2022-03-11

**Authors:** Joseph C. Ahn, Yi-Te Lee, Vatche G. Agopian, Yazhen Zhu, Sungyong You, Hsian-Rong Tseng, Ju Dong Yang

**Affiliations:** 1 Division of Gastroenterology and Hepatology, Mayo Clinic, Rochester, MN 55905, USA.; 2 California NanoSystems Institute, Crump Institute for Molecular Imaging, University of California, Los Angeles, CA 90095, USA.; 3 Department of Molecular and Medical Pharmacology, University of California, Los Angeles, CA 90095, USA.; 4 Department of Surgery, University of California, Los Angeles, CA 90095, USA.; 5 Jonsson Comprehensive Cancer Center, University of California, Los Angeles, CA 90048, USA.; 6 Samuel Oschin Comprehensive Cancer Institute, Cedars-Sinai Medical Center, Los Angeles, CA 90048, USA.; 7 Departments of Surgery, Cedars-Sinai Medical Center, Los Angeles, CA 90048, USA.; 8 Karsh Division of Gastroenterology and Hepatology, Cedars-Sinai Medical Center, Los Angeles, CA 90048, USA.; 9 Comprehensive Transplant Center Cedars-Sinai Medical Center, Los Angeles, CA 90048, USA.

**Keywords:** Hepatocellular carcinoma, surveillance, cirrhosis, hepatitis B virus, alpha-fetoprotein, liquid biopsy, under-utilization

## Abstract

Hepatocellular carcinoma (HCC) is among the leading causes of cancer incidence and mortality worldwide. Surveillance of individuals with cirrhosis or other conditions that confer a high risk of HCC development is essential for early detection and improved overall survival. Biannual ultrasonography with or without alpha-fetoprotein is widely recommended as the standard method for HCC surveillance, but it has limited sensitivity in early disease and may be inadequate in certain individuals. This review article will provide a comprehensive overview of the current landscape of HCC surveillance, including the rationale and indications for HCC surveillance, standard methods for HCC surveillance, and their strengths/limitations. Alternative surveillance methods such as the role of cross-sectional imaging, emerging circulating biomarkers, as well as the problem of under-utilization of HCC surveillance and surveillance-related harms will also be discussed in this review.

## INTRODUCTION

Liver cancer is among the leading causes of global cancer incidence and is the second-most common cause of cancer mortality^[1,2]^. Hepatocellular carcinoma (HCC) is the most dominant form of primary cancer, accounting for roughly 80% of all cases of liver cancer, and occurs in patients with chronic liver diseases of various causes^[3]^. The burden of HCC has historically been high in Asia, with China accounting for over 51% of the global liver cancer burden^[4]^. While a recent analysis has projected a decrease in the incidence and mortality rates of liver cancer in China through 2030, it also emphasized that the overall disease burden would still be serious, especially in rural and western areas due to the persistently high prevalence of HBV and HCV, as well as aflatoxin and alcohol-related liver diseases^[5]^. In the Western world, the HCC incidence rates remain elevated due to the rising prevalence of cirrhosis due to non-alcoholic steatohepatitis (NASH)^[6]^ and alcohol-related liver diseases^[7]^. While there have been significant advances in the treatment of HCC, the overall prognosis in patients with large tumor burden, vascular invasion, or extrahepatic spread continues to remain extremely poor^[8,9]^. Therefore, surveillance of high-risk patients is critical for the detection of HCC at earlier stages where curative options are still available. This review article will provide a comprehensive overview of the indications and rationale for HCC surveillance, currently available surveillance methods and their utilization, effectiveness, and limitations, as well as novel surveillance tools which are emerging as alternatives.

### RATIONALE FOR HCC SURVEILLANCE

The stage at cancer diagnosis is the most important factor determining overall survival in patients with HCC^[10]^. Patients with small, localized tumors may receive curative treatments such as resection, ablation, or liver transplantation, and their long-term survival can be excellent^[11]^. On the other hand, patients with large, multifocal tumors, macrovascular invasion, or extrahepatic metastasis can only receive palliative treatments and have significantly poorer survival despite the advent of newer locoregional and systemic treatment options^[12]^. Therefore, the goal of a surveillance program for HCC is to diagnose tumors at early stages in high-risk individuals so that they can receive curative treatments and achieve improved long-term survival, translating into a decrease in liver cancer mortality^[9]^.

In principle, a randomized controlled trial (RCT) is the best study design to evaluate the effectiveness of medical intervention. However, there has been a paucity of properly conducted, universally generalizable RCTs on the benefits of HCC surveillance. Only two RCTs have been reported, both of which were single-center studies from China in patients with chronic viral hepatitis^[13,14]^. While one of the studies showed a significantly reduced mortality rate in the screening group compared to the control group (HR = 0.63, 95%CI: 0.41–0.98)^[14]^, the other study did not show a difference in mortality (HR = 0.83, 95%CI: 0.68–1.03)^[13]^. In addition, neither study offered their patients the option of choosing nonrandomized screening, and neither study provided information on individual informed consent or contemporary local clinical practice, creating an ethical concern. In 2011, Poustchi *et al.*^[15]^ tested the feasibility of conducting an RCT of HCC surveillance in patients with cirrhosis, and concluded that an RCT of HCC surveillance is not feasible, as most patients decline randomization and prefer to receive surveillance when provided with informed consent.

Despite the absence of large-scale, generalizable RCT data, there is a clear association between receipt of HCC surveillance and improved outcomes. In general, surveillance is considered beneficial to an individual if it provides an increase in life expectancy of around 100 days^[16]^, and cost-effective if surveillance costs < $50,000 per year of life gained^[17]^. A meta-analysis of 47 studies with 15,158 patients demonstrated that HCC surveillance was associated with significantly higher rates of early detection [odds ratio (OR) = 2.08, *P* < 0.01], curative treatments (OR = 2.24, *P* < 0.01), and prolonged survival (OR = 1.90, *P* < 0.01) even after adjusting for lead-time bias^[18]^. Lin *et al.*^[19]^ evaluated the cost-effectiveness of HCC surveillance using a Markov model and determined that ultrasound and alpha-fetoprotein (AFP) was effective with a cost-effectiveness ratio < $50,000 per quality-adjusted life-year. A multistate Markov model simulating a cohort of 50-year-old patients with cirrhosis demonstrated that HCC surveillance decreases all-cause and tumor-specific mortality in patients with compensated cirrhosis regardless of the availability of liver transplantation^[20]^.

### INDICATIONS FOR HCC SURVEILLANCE

Table 1 provides a summary of the latest HCC surveillance recommendations from the American Association for the Study of Liver Diseases (AASLD)^[21]^, European Association for the Study of the Liver (EASL)^[22]^, and Asian Pacific Association for the Study of the Liver (APASL)^[23]^. Patients with cirrhosis have HCC incident rates ranging from 1% to 8% annually and comprise more than 80% of newly diagnosed HCC cases^[21]^. Therefore, all major guidelines recommend HCC surveillance in patients with cirrhosis, except in patients with transplant-ineligible Child-Pugh class C cirrhosis, as HCC surveillance will not provide survival benefit for them due to the competing risk of death from liver failure^[21–23]^.

Another high-risk group includes patients with chronic HBV (CHB) infection. In addition to causing chronic inflammation and fibrosis in the liver, HBV integrates its genome into hepatocyte DNA and induces genetic damage, and can promote carcinogenesis even in the absence of cirrhosis^[24]^. While HCC incidence in CHB patients without cirrhosis is generally lower than in patients with cirrhosis, demographic factors such as age, sex, race/ethnicity, and family history of HCC have been associated with a higher risk of developing HCC. AASLD and APASL recommend HCC surveillance in CHB patients who meet the following criteria: Asian men over 40 years of age; Asian women over 50 years of age; African or African-American; family history of HCC^[21,23]^. EASL guideline utilizes a classification system called PAGE-B (platelet, age, gender, hepatitis B) to define high-risk patients with CHB who would benefit from surveillance^[22,25]^.

While chronic HCV (CHC) infection usually does not cause HCC in the absence of cirrhosis, studies have found that non-cirrhotic CHC patients with high scores in noninvasive markers of fibrosis such as APRI (> 2.0) and FIB-4 (> 4.5) can develop HCC^[26]^. Therefore, the most recent Chinese clinical guidelines for the management of HCC recommend screening all non-cirrhotic patients with CHB as well as chronic HCV infections^[27]^.

The burden of NAFLD-related HCC is rapidly increasing in the United States. While NAFLD patients without cirrhosis have less than 1% annual risk of developing HCC, the high prevalence of NAFLD may still lead to a substantial number of HCC cases among NAFLD patients without cirrhosis. As a result, the most recent practice guidance from the American Gastroenterological Association recommends considering HCC surveillance in patients with NAFLD whose noninvasive markers suggest evidence of advanced liver fibrosis^[28]^.

### UNDER-UTILIZATION OF HCC SURVEILLANCE

The effectiveness of HCC surveillance at improving outcomes in high-risk patients relies not only on the accuracy of surveillance tests but also on the real-life implementation of surveillance. Both provider and patient-related factors can lead to suboptimal adherence to surveillance recommendations. In 2015, a survey of 131 primary care providers at a large the United States urban hospital reported that 65% of the providers reported ordering annual surveillance and only 15% reported ordering biannual surveillance, many incorrectly believing that clinical examination, liver enzymes, or AFP alone without ultrasound were effective surveillance tools^[29]^. Another survey of 391 the United States primary care providers conducted in the same year also showed that only 45% reported ordering HCC surveillance for their patients^[30]^. In addition, many patients have barriers to HCC surveillance due to difficulties in scheduling, cost, and transportation^[31]^. Among the patients surveyed about their knowledge of HCC surveillance, a significant proportion believed that eating a healthy diet would preclude the need for HCC surveillance, or that HCC surveillance was not necessary if they had no symptoms^[31]^. The rates of HCC surveillance are also lower among racial/ethnic minorities and those of lower socioeconomic status^[32]^. A recently published systemic review and meta-analysis of 29 studies showed a pooled estimate for surveillance in 24% of patients, with striking differences in surveillance rate between patients seen in gastroenterology/hepatology clinics and patients in population-based cohorts (73.7% *vs.* 8.8%, *P* < 0.001)^[33]^. Compared to other etiologies of liver disease, patients with NASH or alcohol-associated cirrhosis were less likely to receive surveillance. Interventions including patient/provider education, reminder and recall systems, and population health outreach were identified as effective strategies to improve HCC surveillance rates^[33]^.

Under-utilization of HCC surveillance is also a major health concern in East Asian countries despite the presence of jurisdictional surveillance programs. In 2017, a study of high-risk Chinese patients with CHB and CHC found that only 50% received routine screening for HCC, while 26.7% had incomplete or no screening. Determinants for adherence included a higher level of education, underlying liver cirrhosis, family history of HCC, better knowledge of the disease, while common barriers included lack of awareness (41.5%), absence of symptoms (38.3%), and lack of recommendation from physicians (31.9%)^[34]^. Another recent study of high-risk patients in Yunnan, China, found that of a total of 327 patients who were eligible for HCC surveillance, only a quarter of them underwent HCC surveillance within 7 months. High costs and perceived poor test efficacy were two major barriers, and knowledge of HCC surveillance and lifestyle also had an influence on adherence to HCC surveillance^[35]^. Similarly, a South Korean study also showed that the surveillance rate among high-risk patients was only 40% despite the presence of a national guideline. Only 57.1% of the patients were aware of the need for regular surveillance, and less than half (44.2%) of the patients had accurate information about the surveillance method^[36]^.

### POTENTIAL HARMS OF SURVEILLANCE

Clinicians must consider not only the obvious benefits of HCC surveillance, but its potential harms, which can be physical, financial, as well as psychological. A false positive or indeterminate surveillance result may cause physical harm by subjecting the patient to repeated exposure to contrast agents or ionizing radiation, or even an invasive liver biopsy. Financial harms come from the costs of screening and diagnostic evaluation, as well as transportation and days of missed work. Finally, repeatedly being reminded of one’s risk of developing deadly cancer and waiting to be told the verdict every six months can be a significant emotional burden and lead to fear, anxiety, and depression. Fortunately, studies of surveillance-related harms to date have shown that the harms of HCC surveillance are mild for the vast majority of patients and that the overall benefits of HCC surveillance greatly outweigh its harms^[37–39]^. However, it must be noted that most of the studies focused on physical harms, and more studies are needed to properly assess the burden of financial and psychological harms from HCC surveillance.

### CURRENTLY ACCEPTED HCC SURVEILLANCE METHODS

#### Standard surveillance using ultrasound with or without alpha-fetoprotein

Liver ultrasonography every 6 months with or without serum AFP level is widely recommended as the standard modality for HCC surveillance [Table 2]^[21–23]^. The main advantages of ultrasound include its widespread availability, low cost, and noninvasiveness^[40]^. There have been debates surrounding the effectiveness of ultrasound-based surveillance, as the quality of liver ultrasound can be limited by various factors, including sex, body habitus, and the underlying etiology of liver disease. A study of 941 patients who received ultrasound for HCC surveillance identified male gender, body mass index, Child-Pugh class B or C cirrhosis, alcohol-related cirrhosis, NASH cirrhosis, and inpatient status as independent risk factors for inadequate ultrasound quality^[41]^. A recent meta-analysis concluded that up to 21.5% of ultrasounds done for HCC surveillance were inadequate^[42]^. Another meta-analysis of 32 studies, including 13,367 patients, demonstrated that ultrasound had an 84% sensitivity for detection of HCC at all stages but only had a 47% sensitivity for early-stage HCC^[43]^. However, this study was limited by aggregating all of the studies from the pre-2000s with studies done after the 2000s, when the advent of harmonic and compound imaging significantly increased ultrasound’s sensitivity for detection of early HCC^[44]^. Indeed, when the results were analyzed by the decade of publication, ultrasound’s sensitivity for early-stage HCC increased from 29.7% in the 1990s to 62.2% in the 2010s^[45]^. Several recent studies reported that ultrasound with or without AFP has a sensitivity ranging between 69%−88% for early-stage HCC^[46–49]^.

Contrast-enhanced ultrasound (CEUS) utilizing microbubble-based contrast materials such as perfluorobutane (Sonazoid) is expected to further increase the efficacy of ultrasound-based HCC surveillance. CEUS improves the assessment of tumor boundaries, tumor vascularity, and tumor characterization compared to B-mode ultrasound^[50]^. In a cohort of 292 patients under HCC surveillance, CEUS was able to detect 16 additional HCCs that were not detected on B-mode ultrasound^[51]^. In a prospective, multicenter study of 23 institutions, the average size of HCCs detected by CEUS was significantly smaller than the size of HCCs detected by B-mode ultrasound, suggesting that CEUS is superior to B-mode ultrasound for early detection^[52]^. Another multicenter study has reported that the use of CEUS for HCC surveillance led to a significantly reduced false positive rate by 23.2%, although it did not lead to improved early detection^[53]^.

Serum AFP has been widely used as a predictive biomarker for HCC and is generally associated with tumor size^[54]^. When used with ultrasound, it has been shown to increase the sensitivity for early-stage HCC^[43]^. However, AFP also has limited sensitivity and specificity for early-stage HCC. For HCC tumors less than 5cm in size, AFP has a sensitivity between 49% to 71% and specificity between 49% to 86%^[55]^. False elevations in AFP can be seen in the setting of active hepatic inflammation (e.g., viral hepatitis) or other liver masses such as cholangiocarcinoma^[54]^. Studies have proposed that the accuracy of AFP in HCC surveillance can be improved by measuring a longitudinal trend rather than a single value and also by accounting for additional factors such as serum alanine aminotransferase level and etiology of liver disease^[56–58]^.

#### CT and MRI in HCC surveillance

Multiphase, contrast-enhanced, cross-sectional imaging modalities such as computerized tomography (CT) or magnetic resonance imaging (MRI) are not recommended as first-line surveillance methods due to their lower availability, higher cost, exposure to radiation (CT) and contrast material (CT and MRI), and poor patient tolerance (MRI)^[9]^. A randomized controlled trial comparing ultrasound and CT for HCC surveillance showed that biannual ultrasonography was marginally more sensitive and less costly for detection of early-stage HCC compared to annual CT scans^[59]^. However, CT or MRI may be indicated as alternatives to ultrasound in patients who are likely to have inadequate ultrasound images or in certain high-risk patients in whom the benefits of contrast-enhanced cross-sectional imaging outweigh their downsides. In a prospective study at a tertiary care center, 407 patients with cirrhosis whose estimated annual risks of HCC were greater than 5% underwent biannual HCC surveillance using paired ultrasound and liver-specific contrast-enhanced MRI. Of the 43 cases of incident HCC diagnosed over the 3 years of study period, MRI detected 37 (86.0%) while ultrasound only detected 12 (27.9%) of the cases^[60]^. It should be noted that this was not a randomized controlled trial and not optimally designed for a head-to-head comparison of the two modalities. In addition, a Markov model in a cohort of high-risk patients with predominantly HBV-related compensated cirrhosis showed that biannual HCC surveillance using liver-specific contrast-enhanced MRI may be more cost-effective compared to ultrasound despite its higher cost^[61]^. Therefore, AASLD recommends utilizing CT or MRI for HCC surveillance in select patients with inadequate ultrasound or those with a high likelihood of having an inadequate ultrasound^[21]^, while EASL recommends using CT or MRI for HCC surveillance in high-risk patients on the liver transplant waiting list [Table 2]^[22]^. To minimize the scanning time, cost, and contrast exposure during MRI, abbreviated MRI protocols have been developed with promising performances in small cohort studies^[62,63]^.

### EMERGING HCC SURVEILLANCE METHODS

#### Novel serum protein-based biomarkers

Given the limitations of AFP, there are ongoing efforts to search for novel blood-based biomarkers of HCC. AFP-L3 is a glycoform of AFP with a high affinity for Lens culinaris agglutinin, and it has shown promises for early detection and prediction of tumor aggressiveness in patients with HCC^[64]^. Des-y-carboxy prothrombin (DCP) is a protein induced by vitamin K deficiency or antagonist-II and has been demonstrated to be a useful diagnostic and prognostic marker of HCC in several studies^[65,66]^. In 2014, a prospective, UK-based single-center study of 670 patients with chronic liver diseases was conducted to identify risk factors and blood-based biomarkers of HCC. The final “GALAD” score consisting of gender, age, AFP-L3, AFP, and DCP provided excellent performances with an area under the receiver operating characteristic curve (AUROC) over 0.90 for prediction of HCC regardless of stage^[67]^. The GALAD score was validated in a large, international cohort of nearly 7000 patients and demonstrated its abilities to discriminate patients with HCC from patients with chronic liver diseases or patients with other hepatobiliary tumors^[68]^. A United States study of patients with cirrhosis or chronic hepatitis B found that the GALAD score significantly outperformed liver ultrasound for detection of early-stage HCC, and that combining the GALAD score with ultrasound could achieve an AUROC of 0.98 with 95% sensitivity and 91% specificity for HCC detection^[69]^. A recent study of patients from Germany and Japan showed that the GALAD score might detect HCC with high accuracy in NASH patients with or without cirrhosis^[70]^. Despite the remarkable performances seen in recent studies, the incorporation of the GALAD model in real-life clinical practice has been slow, and it has not yet been formally endorsed by the major liver societies, and more robust phase 3 biomarker study results are eagerly awaited^[71]^.

#### Liquid biopsy

Liquid biopsy refers to analyzing samples of body fluids to obtain important phenotypic, genetic, and transcriptomic information about the primary tumor^[72]^. Recently, various liquid biopsies, including circulating tumor cells (CTCs), circulating tumor DNA (ctDNA), and extracellular vesicles (EVs), have emerged as novel biomarkers with the potential for implementation of precision medicine in the care of patients with HCC.

CTCs are cancer cells in circulation derived from the original tumor or metastatic foci, representing a noninvasive way of sampling live tumor cells from patients with HCC^[73]^. Compared to other liquid biopsy approaches, CTC is less utilized as a diagnostic biomarker given its low detection rate among patients with early-stage HCC and time-consuming process for enumerating CTCs^[73]^. In 2017, Kalinich *et al.*^[74]^ developed a panel of 10 HCC-specific mRNA markers and demonstrated that measuring these 10 markers in enriched CTCs enabled detecting HCC from healthy donors and chronic liver disease. Although their strategy overcame the hurdle of CTC enumeration, only 56% of all-stage HCC patients were classified correctly.

ctDNA contains DNA from dying or phagocytized tumor cells and can also provide valuable tumor-specific mutations and epigenetic changes in patients with HCC^[75]^. Recently, several assays measuring the methylation level of ctDNA for HCC detection have received Food and Drug Administration breakthrough device designation and Conformité Européene Mark^[76–79]^. However, HCC patients in these studies were not restricted to early-stage disease, which could lead to overestimation of biomarker performance for the purpose of early HCC detection^[80]^.

EVs are microvesicles that transport a range of biological molecules and mediate the communication between HCC tumors and their surrounding environments^[81]^. Similar to ctDNA, EV and its cargoes are regarded as promising biomarkers to augment current HCC surveillance methods due to their early presence in circulation^[81]^. Our recent phase 2 biomarker study applied an HCC EV Digital Scoring Assay to quantify the identified 10 HCC-specific mRNA markers^[74]^ from purified HCC EVs and showed the resultant digital scores could noninvasively detect BCLC stage 0-A HCC from cirrhosis with an AUROC of 0.93 (sensitivity = 94%, specificity = 89%)^[82]^. Currently, almost all of the existing EV-based biomarker studies are still at phase II (case-control)^[81,83]^, further phase III (prospective specimen collection, retrospective blinded evaluation), and phase IV studies (prospective cohort) are required to validate their performance for detecting early-stage HCC^[80]^.

#### The role of artificial intelligence

The recent decade has seen explosive growth in the application of artificial intelligence (AI) in medicine, and the field of hepatology has been no exception to this trend. AI-based machine learning algorithms, including deep learning, can process a wide spectrum of healthcare data from structured numeric data, free texts in medical documentation, high-dimensional data from multi-omics, and digitized high-resolution images from radiologic and histopathologic studies^[84]^. As AI algorithms have the capacity to synthesize and analyze complex relationships within large amounts of data in ways that are impossible for humans, they have great potential to improve risk prediction and diagnostic accuracy in HCC surveillance. Several recently reported machine learning models trained using clinical data from large cohorts of patients with viral hepatitis demonstrated high performances for predicting longitudinal risk of HCC incidence and significantly outperformed conventional prediction models^[85–87]^. Deep learning algorithms utilizing convolutional neural networks have revolutionized computer vision and image processing, and they have been applied to ultrasound^[88–90]^, CT^[91,92]^, and MRI^[93,94]^ images of patients with or without hepatic lesions to detect HCC, sometimes outperforming human radiologists. While there are concerns over the interpretability and universal generalizability of the AI algorithms^[95]^, state-of-the-art AI algorithms are rapidly being applied for the care of patients with HCC and hold great potential to fill the unmet needs in HCC surveillance.

## CONCLUSION

Surveillance in high-risk patients is critical for early detection of HCC, which leads to higher chances of curative treatment and prolonged survival. Biannual ultrasound with or without AFP remains the standard surveillance method endorsed by major societies, but contrast-enhanced cross-sectional imaging may be indicated in patients awaiting liver transplantation or patients with inadequate ultrasound image qualities. Emerging blood-based biomarkers such as the GALAD score and liquid biopsy techniques are promising additional tools for HCC detection. In addition, the rapid developments in AI technology may greatly improve individualized HCC risk prediction and interpretation of imaging studies. Finally, the significant under-utilization of HCC surveillance is a major problem. Widespread patient/provider education and outreach efforts are necessary to make sure the at-risk patients receive the benefits of HCC surveillance while minimizing the potential physical, financial, and psychological harms.

## Figures and Tables

**Table 1.** Indications for HCC surveillance

AASLD	EASL	APASL

1. All adults with cirrhosis, except for Child-Pugh class C patients ineligible for liver transplant	1. Cirrhotic patients, Child-Pugh stage A & B	1. All adults with cirrhosis, except for Child-Pugh class C patients ineligible for liver transplant
	2. Cirrhotic patients, Child-Pugh stage C awaiting LT	
	3. Non-cirrhotic HBV patients at intermediate or high risk of HCC according to PAGE-B* classes for Caucasians	
2. High risk patients with HBV		2. High risk patients with HBV
- Asian men age > 40		- Asian men age > 40
- Asian women age > 50	4. Non-cirrhotic F3 patients based on individual risk assessment	- Asian women age > 50
- African ancestry		- African ancestry age > 20
- Family history of HCC		- Family history of HCC
**AGA:** In addition to above recommendations, patients with non-alcoholic fatty liver disease with noninvasive markers showing evidence of advanced liver fibrosis or cirrhosis should be considered for HCC screening		
**Chinese clinical guidelines:** All patients with cirrhosis, HBV, and HCV		

A total sum of 10 or more indicates intermediate or high risk of hepatocellular carcinoma.

* PAGE-B (platelet, age, gender, hepatitis B) score is calculated using age (16–29 = 0, 30–39 = 2, 40–49 = 4, 50–59 = 6, 60–69 = 8, ≥ 70 = 10), sex (male = 6, female = 0), and platelet count (≥ 200,000/μL= 0, 100,000–199,999/μL = 1, < 100,000/μL = 2). AASLD: American Association for the Study of the Liver Diseases; APASL: Asian Pacific Association for the Study of the Liver; EASL: European Association for the Study of the Liver; HBV: hepatitis B virus; HCC: hepatocellular carcinoma.

**Table 2.** Recommended HCC surveillance methods

AASLD	EASL	APASL

1. Biannual abdominal ultrasonography with or without AFP 2. CT and MRI may be utilized in select patients with a high likelihood of having an inadequate ultrasound or if ultrasound is attempted but inadequate	1. Biannual abdominal ultrasonography 2. AFP not recommended particularly in patients with active liver inflammation 3. MRI/CT can be used for patients on the waiting list for liver transplant 4. MRI/CT can be used for patients who have had inadequate ultrasonography, but their risk and cost make their use in long-term surveillance highly debatable	1. Biannual abdominal ultrasonography with or without AFP 2. Cutoff value of AFP should be set at 200ng/mL when used in combination with ultrasonography 3. Cutoff value of AFP can be set lower with hepatitis virus suppression or eradication
**Chinese clinical guidelines:** Biannual abdominal ultrasonography with AFP		

AASLD: American Association for the Study of the Liver Diseases; AFP: alpha-fetoprotein; APASL: Asian Pacific Association for the Study of the Liver; CT: computerized tomography; EASL: European Association for the Study of the Liver; HCC: hepatocellular carcinoma; MRI: magnetic resonance imaging.
